# Up-regulation of NADPH oxidase-mediated redox signaling contributes to the loss of barrier function in KRIT1 deficient endothelium

**DOI:** 10.1038/s41598-017-08373-4

**Published:** 2017-08-15

**Authors:** Luca Goitre, Peter V. DiStefano, Andrea Moglia, Nicholas Nobiletti, Eva Baldini, Lorenza Trabalzini, Julie Keubel, Eliana Trapani, Vladimir V. Shuvaev, Vladimir R. Muzykantov, Ingrid H. Sarelius, Saverio Francesco Retta, Angela J. Glading

**Affiliations:** 1 Department of Clinical and Biological Sciences, University of Torino, Torino, Italy; 2 Department of Pharmacology and Physiology, University of Rochester, New York, USA; 3 Department of Agriculture, Forest and Food Sciences, Plant Genetics and Breeding, University of Torino, Torino, Italy; 4 Department of Biotechnology, Chemistry and Pharmacy, University of Siena, Siena, Italy; 5 Department of Pharmacology, University of Pennsylvania, Pennsylvania, USA

## Abstract

The intracellular scaffold KRIT1/CCM1 is an established regulator of vascular barrier function. Loss of KRIT1 leads to decreased microvessel barrier function and to the development of the vascular disorder Cerebral Cavernous Malformation (CCM). However, how loss of KRIT1 causes the subsequent deficit in barrier function remains undefined. Previous studies have shown that loss of KRIT1 increases the production of reactive oxygen species (ROS) and exacerbates vascular permeability triggered by several inflammatory stimuli, but not TNF−α. We now show that endothelial ROS production directly contributes to the loss of barrier function in KRIT1 deficient animals and cells, as targeted antioxidant enzymes reversed the increase in permeability in KRIT1 heterozygous mice as shown by intravital microscopy. Rescue of the redox state restored responsiveness to TNF-α in KRIT1 deficient arterioles, but not venules. *In vitro*, KRIT1 depletion increased endothelial ROS production via NADPH oxidase signaling, up-regulated Nox4 expression, and promoted NF-κB dependent promoter activity. Recombinant yeast avenanthramide I, an antioxidant and inhibitor of NF-κB signaling, rescued barrier function in KRIT1 deficient cells. However, KRIT1 depletion blunted ROS production in response to TNF-α. Together, our data indicate that ROS signaling is critical for the loss of barrier function following genetic deletion of KRIT1.

## Introduction

Control of vascular permeability is a function of the endothelial cells that line microvessels (arterioles, capillaries and venules), and which form a reasonably tight and uninterrupted barrier that limits the passage of proteins as large or larger than albumin. Under resting conditions, the permeability of the endothelial barrier to molecules such as albumin is largely representative of the effectiveness of this barrier. Recently, we established that the junctional scaffolding protein KRIT1 regulates the effectiveness of this endothelial barrier^1, 2^.

KRIT1 is an 80 kDa protein with several distinct binding sites for proteins involved in the regulation of cell adhesion, including the small GTPase Rap1. ln particular, it is now well established that the KRIT1-Rap1 complex associates with adherens junctions and is required for endothelial junctional stability *in vitro* ^1^ and *in vivo* ^2, 3^. Indeed, in previous studies we discovered that depletion of KRIT1 reduces binding of β− and p120-catenin to the cytoplasmic tail of VE-cadherin, leading to destabilization of VE-cadherin-mediated cell-cell junctions and a consequent increase in endothelial monolayer permeability *in vitro* ^1, 4^. Heterozygous loss of KRIT1 in humans has been linked to the pathogenesis of Cerebral Cavernous Malformation (CCM), a major cerebrovascular disease of proven genetic origin affecting 0.3–0.5% of the population. CCM is characterized by abnormally enlarged and leaky capillaries which are devoid of normal vessel structural components, such as pericytes and astrocyte foot processes, but surrounded by a thickened collagenous matrix^5^. CCM is a debilitating disease for which no non-surgical intervention is currently recommended. Several mouse models have strongly suggested that this disease arises due to defects in endothelial cell-cell contacts^6, 7^ that lead to increased permeability in the brains of patients^8^, contribute to chronic and acute hemorrhage, and lead to chronic neurological symptoms^5, 9^.

We have observed a similar increase in vascular permeability in an intact KRIT1-deficient mouse microvessel network using intravital microscopy^2^. Furthermore, while we showed that KRIT1 heterozygous animals exhibit increased sensitivity to auto-antigens and foreign polysaccharides^2^, our previous studies also revealed an unusual lack of responsiveness to the pro-inflammatory cytokine tumor necrosis factor-alpha (TNF-α), which was not due to an intrinsic change in TNF−α receptor stimulation^2^. Specifically, treatment of KRIT1 heterozygous animals with TNF-α failed to increase vascular permeability, though it did stimulate leukocyte adhesion and extravasation, and other stimuli (e.g. histamine) were able to increase permeability^2^. TNF-α is a major inflammatory stimulus, and a critical target for treatment of many chronic inflammatory diseases. TNF-α stimulates several downstream pathways in the endothelium— including the activation of nuclear factor-κB (NF-κB)–mediated transcription of pro-inflammatory genes— to promote oxidative stress, the adhesion and extravasation of immune cells, and increased permeability of the endothelial barrier^10, 11^. Despite the apparent role for KRIT1 in the regulation of vascular permeability, we still do not have a clear understanding of how KRIT1 regulates changes in cell behavior and vessel homeostasis. Our finding that KRIT1-depleted cells are insensitive to TNF-α —yet respond to other stimuli, suggests that reduced KRIT1 expression does not merely reduce barrier function *in vivo* but actively modifies signaling responses to inflammatory mediators.

Multiple pathways contribute to changes in endothelial phenotype following loss of KRIT1 expression. We noted that many of these pathways contribute to the regulation of cellular reactive oxygen species (ROS) production, which we previously reported as a downstream consequence of loss of KRIT1^12^. ROS production in endothelial cells contributes to the regulation of vascular tone, angiogenesis, and the inflammatory response. Over-production of ROS contributes to endothelial dysfunction, particularly in pathological conditions marked by inflammation and increased permeability^13, 14^. Production of ROS is increased by many stimuli, including cytokines such as TNF-α^15, 16^, thus dysregulation of ROS could explain both the increased basal permeability and the lack of TNF−α-induced permeability in KRIT1-depleted endothelium. However, the contribution of increased ROS to the phenotype of KRIT1-depleted endothelial cells and the pathophysiology of CCM remains unknown.

ROS are produced by the activity of a wide array of cellular enzymes, including NADPH oxidases (Nox), enzymes of the mitochondrial respiratory chain, xanthine oxidases, cytochrome p450 monooxygenases, uncoupled nitric oxide synthase, lipoxygenases, and cyclooxygenases, which can be induced by a variety of endogenous and exogenous chemical and physical stimuli^17^. As mentioned above, our original findings demonstrated an increase in ROS production in KRIT1 depleted cells, showing for the first time that KRIT1 may exert a protective role against oxidative stress events^12^. Indeed, in KRIT1 null mouse embryonic fibroblasts (MEF) and KRIT1 siRNA transfected human umbilical vein endothelial cells loss of KRIT1 expression increased the production of ROS and reduced expression of the mitochondrial ROS scavenger, superoxide dismutase 2 (SOD2), promoting oxidative stress-mediated molecular and cellular dysfunctions^12^. Furthermore, the up-regulation of ROS led to increased expression and activation of the redox-sensitive transcription factor c-Jun, as well as induction of its downstream target cyclooxygenase-2 (COX-2)^18^, suggesting that KRIT1 can limit both pro-oxidant and pro-inflammatory pathways that in turn may influence CCM disease pathogenesis.

Here we show that ROS play a key role in increasing vascular permeability in KRIT1-deficient mice, as antioxidant delivery prevented the increase in baseline permeability in these animals, blocked TNF-α induced increased permeability in venules, and restored the responsiveness of KRIT1-deficient arterioles to TNF-α. Furthermore, these ROS-dependent effects were associated with the up-regulation of NADPH oxidases, specifically Nox4, and could be reversed by N-(E)-p-coumaroyl-3-hydroxyanthranilic acid (Yeast avenanthramide I, YAv1)^19^, a novel antioxidant and inhibitor of NF-κB signaling, revealing a novel mechanism of crosstalk between the adherens junction and inflammatory signaling.

## Results

### Endothelial-targeted antioxidant enzymes reverse increased baseline microvessel permeability in KRIT1 deficient mice

Our previous studies demonstrated that KRIT1 hemizygous (Krit1^+/−^) mice exhibit a 2-fold increase in microvessel permeability, in both arterioles and venules^2^. This difference is exaggerated in animals with a complete loss of KRIT1 (data not shown). It is well described that several permeability inducing stimuli, including TNF-α, interleukin-1β (IL-1β), and lipopolysaccharide (LPS), stimulate ROS production and that scavenging ROS can limit permeability *in vitro* and *in vivo*. As it had also been previously reported that loss of KRIT1 expression can increase ROS production^12^, we tested whether scavenging ROS from the endothelium could reverse the increased permeability in KRIT1 deficient mice.

**Figure 1:**
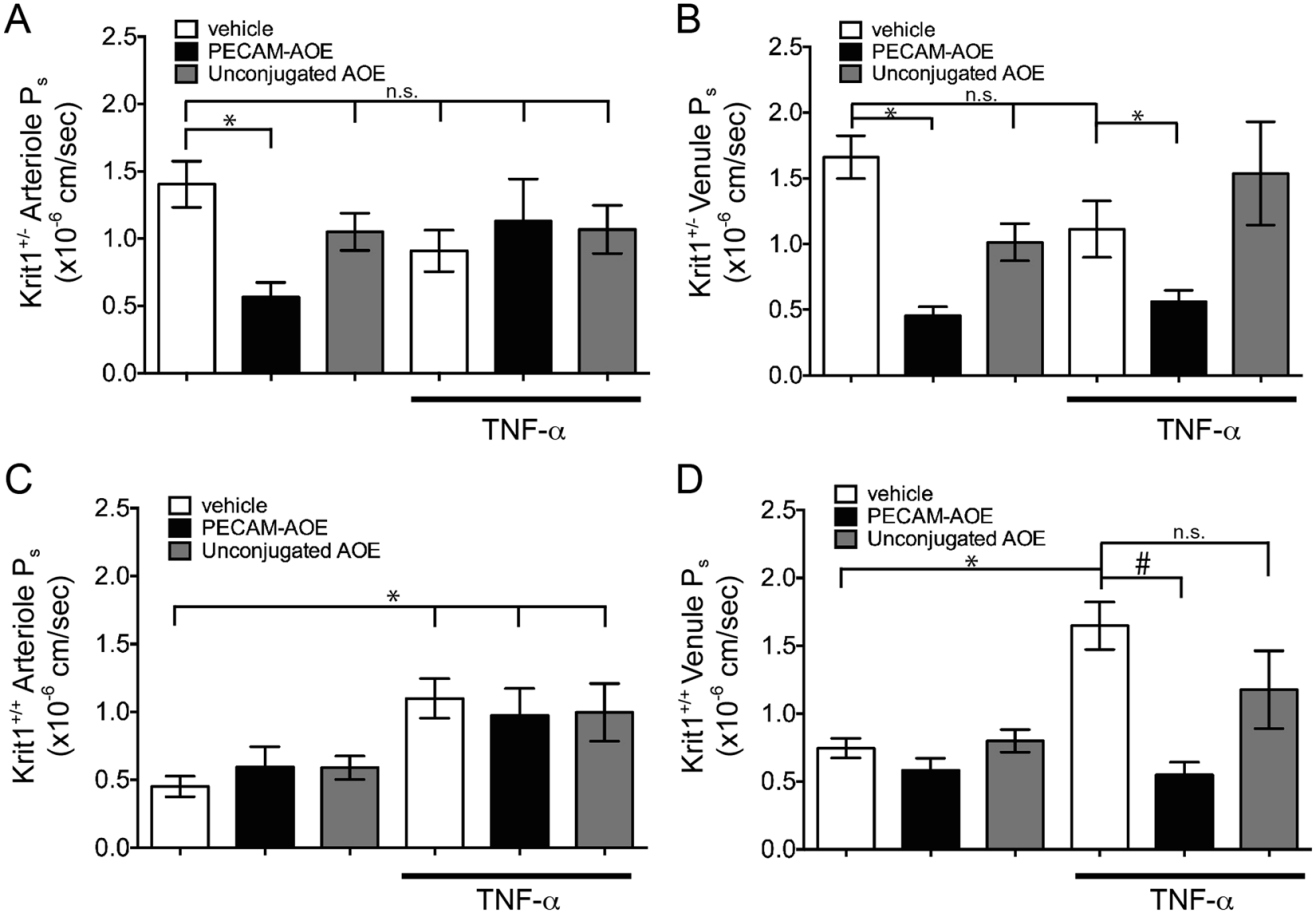
Endothelial-targeted antioxidant enzymes reverse increased micro-vessel permeability in KRIT1 deficient mice. (**A**) Cremaster arteriole permeability in KRIT1 heterozygous (Krit1^+/−^) mice treated with PECAM-AOE, unconjugated AOE, or vehicle alone +/− TNF-α. n = 10 vessel sites; p < 0.001 by non-parametric ANOVA; *p < 0.05 by Dunn’s post-hoc test, n.s.- not significant. (**B**) Cremaster venule permeability under same conditions as in (**A**). p < 0.001 by non-parametric ANOVA; *p < 0.05 by Dunn’s post-hoc test, n.s.- not significant. (**C**) Cremaster arteriole permeability in wild type (Krit1^+/+^) mice treated with PECAM-AOE, unconjugated AOE, or vehicle alone in the presence or absence of TNF-α. Data shown are the mean P_s_ +/− SEM. n = 10 vessel sites; p < 0.001 by non-parametric ANOVA; *p < 0.05 by Dunn’s post-hoc testing.(**D**) Cremaster venule permeability under same conditions as in (**A**). p < 0.001 by non-parametric ANOVA; *p < 0.01 or ^#^p < 0.05 by Dunn’s post-hoc test, n.s.- not significant.

Because vascular abnormalities make intravital permeability measurements in KRIT1 null animals extremely challenging, we elected to treat Krit1^+/−^ mice with a novel antioxidant agent specifically designed to target its delivery and effect to the endothelium. This agent is comprised of two antioxidant enzymes (AOE): superoxide dismutase (SOD), which converts the superoxide radical (O_2_^·−^) into hydrogen peroxide (H_2_O_2_), and catalase, which converts H_2_O_2_ into oxygen and water, each conjugated with an antibody that targets platelet-endothelial cell adhesion molecule-1 (PECAM-1). These conjugates (PECAM-AOE) bind to PECAM-1 on the endothelial cell surface and get internalized^20^. Due to this targeting mechanism, PECAM-AOE quench ROS preferentially in the vascular endothelium^21–23^. Previous studies have validated the distribution and efficacy of these molecules *in vivo* and *in vitro* ^24^; injection of 100 µg of each conjugate was sufficient to inhibit H_2_O_2_ or VEGF-induced endothelial permeability *in vitro* ^25^, and ROS-induced pulmonary edema *in vivo* ^26^. Thus, according to previously optimized and validated *in vivo* experimental conditions, we injected 100 µg of PECAM-AOE via the tail vein into KRIT1 heterozygous (Krit1^+/−^) and wild type (Krit1^+/+^) mice 1hr prior to intravital imaging of Alexa 488-bovine serum albumin (BSA) permeability. Remarkably, as compared with control treatment (vehicle), PECAM-AOE treatment significantly reduced baseline permeability in Krit1^+/−^ arterioles and venules (Fig. 1A,B, PECAM-AOE vs. vehicle: arterioles- 0.56 +/− 0.11 vs. 1.4 +/− 0.17; venules- 0.45 +/− 0.07 vs. 1.56 +/− 0.18), leading to levels equivalent to wild type (Krit1^+/+^) littermates (compare Fig. 1A,B PECAM-AOE with Fig. 1C,D vehicle). In contrast, untargeted AOE (injection of SOD/catalase unconjugated enzymes) did not significantly reduce Krit1^+/−^ baseline vascular permeability (Fig. 1A,B, unconjugated AOE vs. vehicle), supporting previous findings that antibody-mediated targeting of the enzymes increases their efficiency.

### Endothelial-targeted antioxidant enzymes limit TNF-α induced permeability in venules, but not arterioles

We then asked whether antioxidant treatment could affect TNF-α induced permeability in our model. As expected, TNF-α increased both arteriole and venule permeability in wild type (Krit1^+/+^) animals (Fig. 1C,D, vehicle + TNF-α vs. vehicle: arterioles- 1.17 +/− 0.09 vs. 0.59 +/− 0.08, venules- 1.59 +/− 0.15 vs. 0.67 +/− 0.09). In these animals, treatment with PECAM-AOE, but not unconjugated AOE, reversed TNF-α induced permeability in venules (Fig. 1D, PECAM-AOE + TNF-α, 0.55 +/− 0.09), but had no effect in arterioles (Fig. 1C, PECAM-AOE + TNF-α, 1.14 +/− 0.15).

On the other hand, as we reported previously^2^, TNF-α treatment was unable to increase permeability in Krit1^+/−^ mice (Fig. 1A,B, vehicle + TNF-α vs. vehicle: arterioles- 0.91 +/− 0.15 vs. 1.4 +/− 0.17; venules- 1.11 +/− 0.21 vs. 1.56 +/− 0.18). Furthermore, we found that PECAM-AOE treatment reversed the increased permeability in TNF-α treated venules (Fig. 1B, PECAM-AOE + TNF-α, 0.56 +/− 0.09), but not in TNF-α treated arterioles (Fig. 1A, PECAM-AOE + TNF-α, 1.13 +/− 0.31), despite the fact that it did inhibit permeability in Krit1^+/−^ arterioles not treated with TNF-α (Fig. 1A, PECAM-AOE + vehicle vs. vehicle). Thus, in Krit1^+/−^ arterioles, it appears that limiting ROS can restore the permeability response to TNF-α, suggesting that a component of the TNF-α–driven ROS production pathway is affected by the loss of KRIT1.

### Loss of KRIT1 blunts TNF-α-induced increased ROS

**Figure 2:**
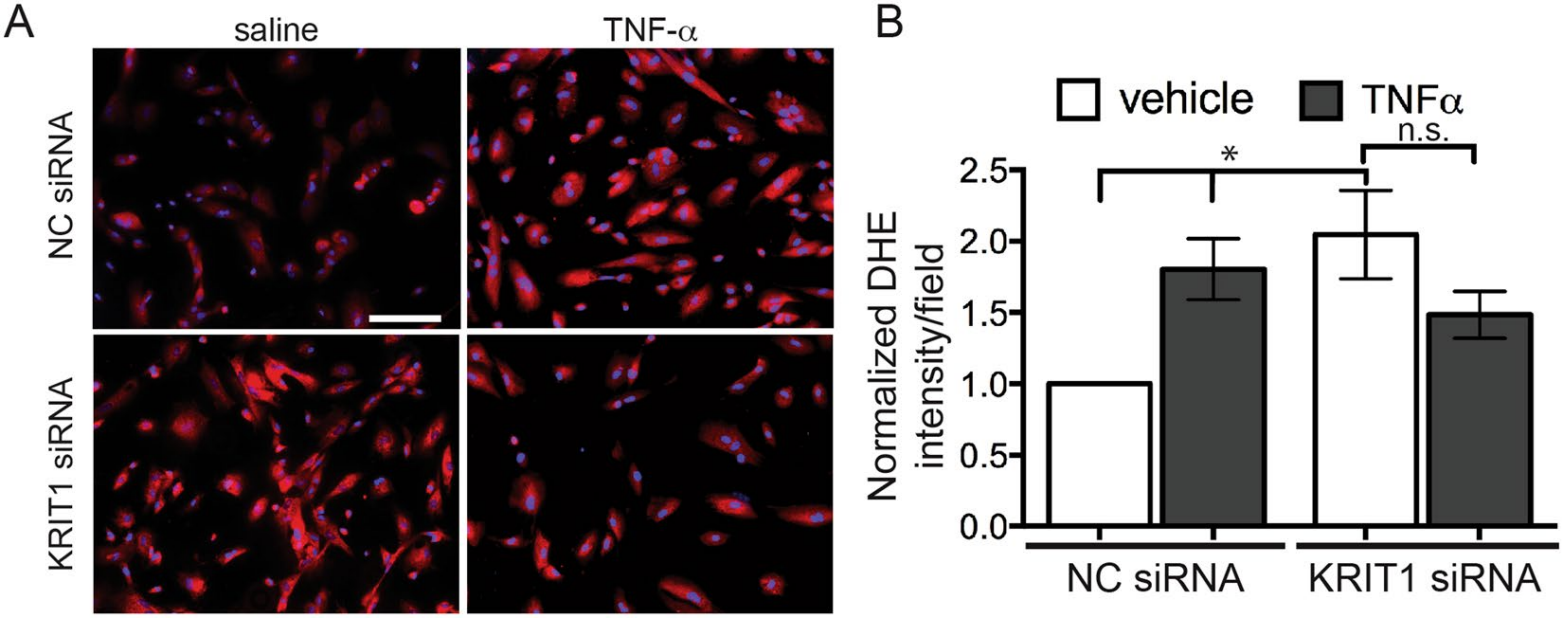
Loss of KRIT1 blunts TNF-α-induced increased ROS. (**A**) DHE fluorescence in negative control (NC) siRNA or anti-KRIT1 siRNA transfected HPAEC treated +/− 25 ng/ml TNF-α. Images are representative of n = 4 independent experiments. (**B**) Average DHE intensity per field of view +/− SEM, normalized to NC siRNA + vehicle, n = 16. p = 0.0137 by ANOVA; *p < 0.05 by Tukey’s post-hoc test, n.s., not significant.

Increased ROS production downstream of TNF-α stimulation is known to mediate changes in vascular permeability, gene expression, and other cell and tissue responses^27, 28^. Given that the absence of KRIT1 caused high baseline levels of ROS, we asked whether this could potentially explain why KRIT1-deficient cells and animals have an abnormal response to TNF-α. Dihydroethidium (DHE) staining, a fluorescent indicator of oxidation, was used to detect ROS in KRIT1 siRNA-transfected cells treated with and without 100 ng/ml TNF-α. As previously reported, knockdown of KRIT1 in human arterial endothelial cells, and treatment of negative control (NC)-siRNA transfected cells with TNF-α, increased DHE staining. However, treatment of KRIT1-depleted cells with TNF-α was unable to increase DHE staining (KRIT1 siRNA, 2.05 +/− 0.31 vs. KRIT1 siRNA + TNF−α, 1.48 +/− 0.16, Fig. 2A,B). These data suggest that in the absence of KRIT1, TNF-α is unable to stimulate ROS production, which may contribute to the lack of permeability response.

### Loss of KRIT1 increases production of ROS via up-regulation of Nox4

**Figure 3:**
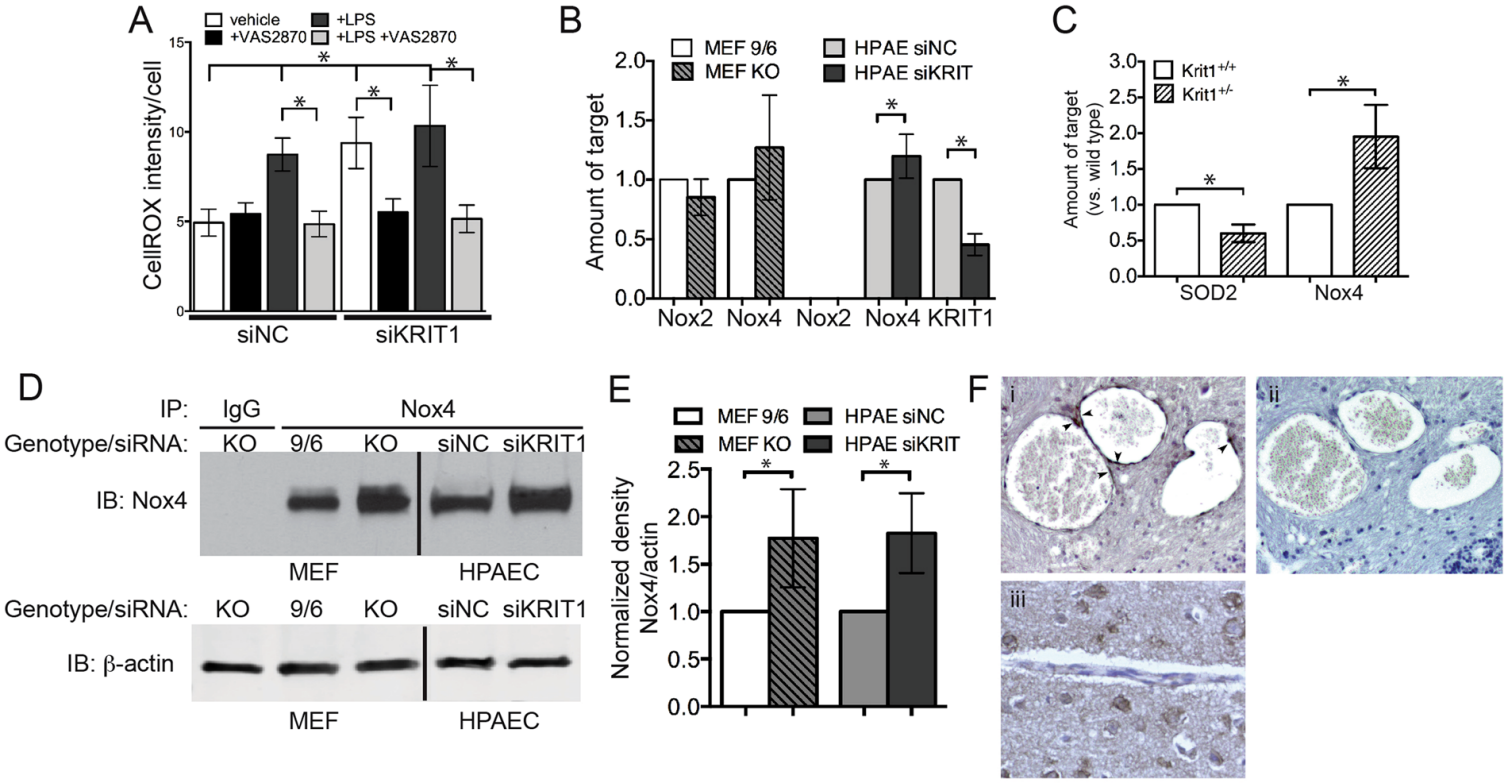
Loss of KRIT1 increases production of ROS via up-regulation of Nox4. (**A**) CellROX®Green fluorescence in negative control (NC) and KRIT1 siRNA transfected HPAEC treated with the Nox inhibitor VAS2870, +/−1 µg/mL LPS. Data are mean +/− SEM, p < 0.001 by ANOVA; *p < 0.05 using Dunn’s post-hoc test, n = 11. (**B**) Relative mRNA expression in KO and reconstituted (9/6) MEF, and NC or KRIT1 siRNA transfected HPAEC. Data are mean +/− SEM, n = 6. Nox2 levels were undetectable in HPAEC. p < 0.001 by ANOVA; *p < 0.05 by Tukey’s post-hoc test. (**C**) Relative mRNA expression in wild type (Krit1^+/+^) and KRIT1 heterozygous (Krit1^+/−^) mouse lung tissue. p < 0.001 by ANOVA; *p < 0.01 by Tukey’s post-hoc test, n = 6. (**D**) Nox4 protein expression in KO and 9/6 MEF, and in NC and KRIT1 siRNA transfected HPAEC. Nox4 was immunoprecipitated from 500 µg total cell protein for each condition. Whole cell lysate was probed for beta-actin as loading control. Representative blot was cropped for space considerations; vertical line indicates intervening lanes removed, n = 4. (**E**) Densitometry analysis of (**D**). Expression of Nox4 normalized to loading control beta-actin, p < 0.001 by ANOVA; *p < 0.05 by Tukey’s post-hoc test. (**F**) Nox4 expression in CCM-like lesions of Krit1^ECKO^ mice. (i) Nox4 staining (dark brown, arrowheads) lines the cavernous lesions of a CCM-like lesion in the midbrain; (ii) Serial section of the same lesion as in (i), stained with rabbit IgG as a negative control; (iii) Normal vessel in cerebellum of Krit1^flox/+^ mouse (Krit1^+/−^). Images are representative, n = 6 mice/group.

Endothelial cells contain several sources of ROS, of these, the NADPH oxidases (Nox) are common targets of cytokine and chemokine signaling, can be regulated by molecules that also regulate cell adhesion, and have a well described role in vascular pathophysiology^16^. We therefore tested whether NADPH oxidases were involved in the increased ROS production in KRIT1 depleted cells by treating KRIT1-siRNA transfected cells with the pan-Nox inhibitor VAS2870. Ten µM VAS2870 reduced ROS levels in KRIT1 siRNA transfected cells to control levels (siKRIT1 +veh, 9.38 +/− 1.43 vs. siKRIT1 +VAS2870, 5.5 +/− 0.76, Fig. 3A), as shown by reduced fluorescence intensity per cell of the ROS sensor CellROX®Green. This was similar in magnitude to the effect of this inhibitor on LPS-induced CellROX staining, which is known to be driven by Nox enzymes, strongly suggesting that loss of KRIT1 increases the activity of one or more Nox isoforms.

We measured Nox2 and Nox4 mRNA expression in both KRIT1 deficient (KO) and in KRIT1 overexpressing (9/6) MEFs (Fig. 3B) and found that loss of KRIT1 expression did not significantly effect Nox2 or Nox4 mRNA expression. In KRIT1 siRNA-transfected human pulmonary artery endothelial cells (HPAEC), Nox2 levels were undetectable, while Nox4 mRNA levels were modestly higher compared to the control (HPAEC siKRIT1 1.2 +/− 0.1 vs. HPAEC siNC 1.0 +/− 0.05, Fig. 3B). To examine Nox expression in wild type and KRIT1 heterozygous animals, we isolated RNA from mouse lung tissue, which is enriched in endothelial cells. Nox4 mRNA was strongly up-regulated in KRIT1 (Krit1^+/−^) heterozygous tissue, while SOD2 expression was slightly lowered (Fig. 3C). These data suggest that Nox4 mRNA expression is affected by loss of KRIT1 in a cell-type specific manner.

We then examined Nox4 expression at the protein level. Indeed, Nox4 protein and activity levels are tightly correlated, as this enzyme is constitutively activated. As shown in Fig. 3D,E, Nox4 protein was strongly up-regulated in KRIT1 null MEFs (KO) and KRIT1 siRNA transfected (siKRIT1) HPAEC. Taking advantage of endothelial KRIT1 null (*Krit1* ^*ECKO*^) and heterozygous (*Krit1* ^+/−^) mice derived from a conditional endothelial-specific KRIT1 knockout mouse model (PDGFBiCreER^T2^Krit1^flox/flox 6^, see Methods), we also found that Nox4 was up-regulated in the endothelium of CCM-like lesions in *Krit1* ^*ECKO*^ animals (Fig. 3F, i), as compared to non-lesion vessels (not shown), or to heterozygous controls (*Krit1* ^+/−^, Fig. 3F, iii). Taken together, these data strongly suggest that the loss of KRIT1 causes a significant up-regulation of this constitutive, Nox4-mediated, pro-oxidant pathway, both *in vitro* and *in vivo*.

### Loss of KRIT1 promotes NF-κB signaling

**Figure 4:**
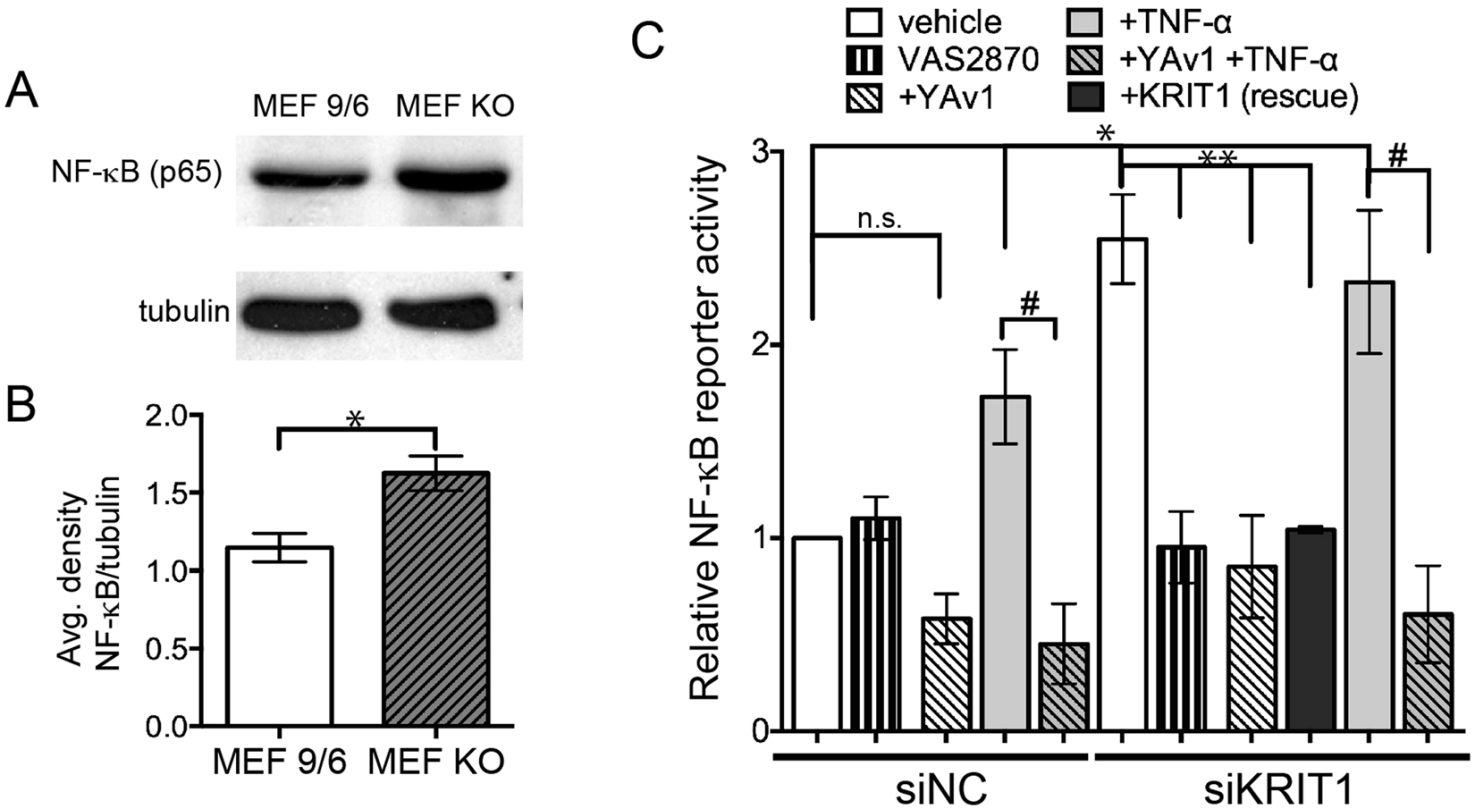
Loss of KRIT1 promotes NF-κB signaling. (**A**) NF-κB p65 expression in lysates of KRIT1 null (KO) and reconstituted (9/6) MEFs. Tubulin is shown as loading control. Representative blots were cropped for space considerations, n = 5. (**B**) Densitometry analysis of (**A**). Expression of NF-κB normalized to loading control tubulin, *p < 0.05 by two-tailed t-test. (**C**) NF-κB reporter activity in negative control (NC) and anti-KRIT1 siRNA transfected HPAEC treated with 10 µM VAS2870, 100 µM YAv1, 25 ng/ml TNF-α, or both YAv1 and TNF-α. KRIT1 (rescue) = cells transfected with KRIT1 siRNA and a rescue plasmid containing a silent mutation of the siRNA target sequence. Data shown are normalized to vehicle-treated siNC. p < 0.0001 by ANOVA; using Tukey’s post-hoc test *p < 0.05 vs. siNC vehicle, **p < 0.05 vs. siKRIT1 vehicle, ^#^p < 0.05 vs. TNF-α treatment, n.s.- not significant, n = 5.

In a previous study, we examined whether the NF-κB-dependent up-regulation of intercellular adhesion molecule-1 (ICAM-1) was affected by the loss of KRIT1 expression and found a slight increase in NF-κB reporter activity in KRIT1 depleted cells, but no significant increase in ICAM-1 expression^2^. As inflammatory stimuli and ROS commonly activate NF-κB transcriptional activity, we reexamined the activation of NF-κB signaling in KRIT1 depleted cells. KRIT1 null MEFs (KO) expressed higher levels of NF-κB p65 compared to MEFs expressing KRIT1 (MEF 9/6, Fig. 4A,B), suggesting that the loss of KRIT1 may up-regulate NF-κB signaling. In addition, depletion of KRIT1 in HPAEC caused a 2.5-fold increase in NF-κB luciferase reporter activity compared to the control (siKRIT1 + vehicle, 2.55 +/− 0.23 vs. siNC + vehicle, Fig. 4C). This activity was reversed by re-expression of a siRNA resistant KRIT1 ( + KRIT1 (rescue), 1.04 +/− 0.02, Fig. 4C). KRIT1 depletion-dependent NF-κB activity could also be blocked by treatment with the pan-Nox inhibitor VAS2870 suggesting that the up-regulation of NF-κB signaling in KRIT1-depleted cells is dependent on Nox activity (siKRIT1 + veh, 2.55 +/− 0.23 vs. siKRIT1 + VAS2870, 0.95 +/− 0.19, Fig. 4C). In addition, the recently described antioxidant N-(E)-p-coumaroyl-3- hydroxyanthranilic acid (Yeast avenanthramide I, YAv1) inhibited both KRIT1 depletion-dependent NF-κB activity (siKRIT1 + veh, 2.55 +/− 0.23 vs. siKRIT1 + YAv1, 0.85 +/− 0.26) and TNF-α induced NF-κB activity (compare siNC + TNF−α vs. siNC + YAv1 + TNF−α, and siKRIT1 + TNF−α vs. siKRIT1 + YAv1 + TNF−α, Fig. 4C), suggesting that this compound blocks a common mechanism of NF-κB activation.

### Increased permeability in KRIT1 deficient cells and animals can be reversed by N-(E)-p-coumaroyl-3- hydroxyanthranilic acid (YAv1)

**Figure 5:**
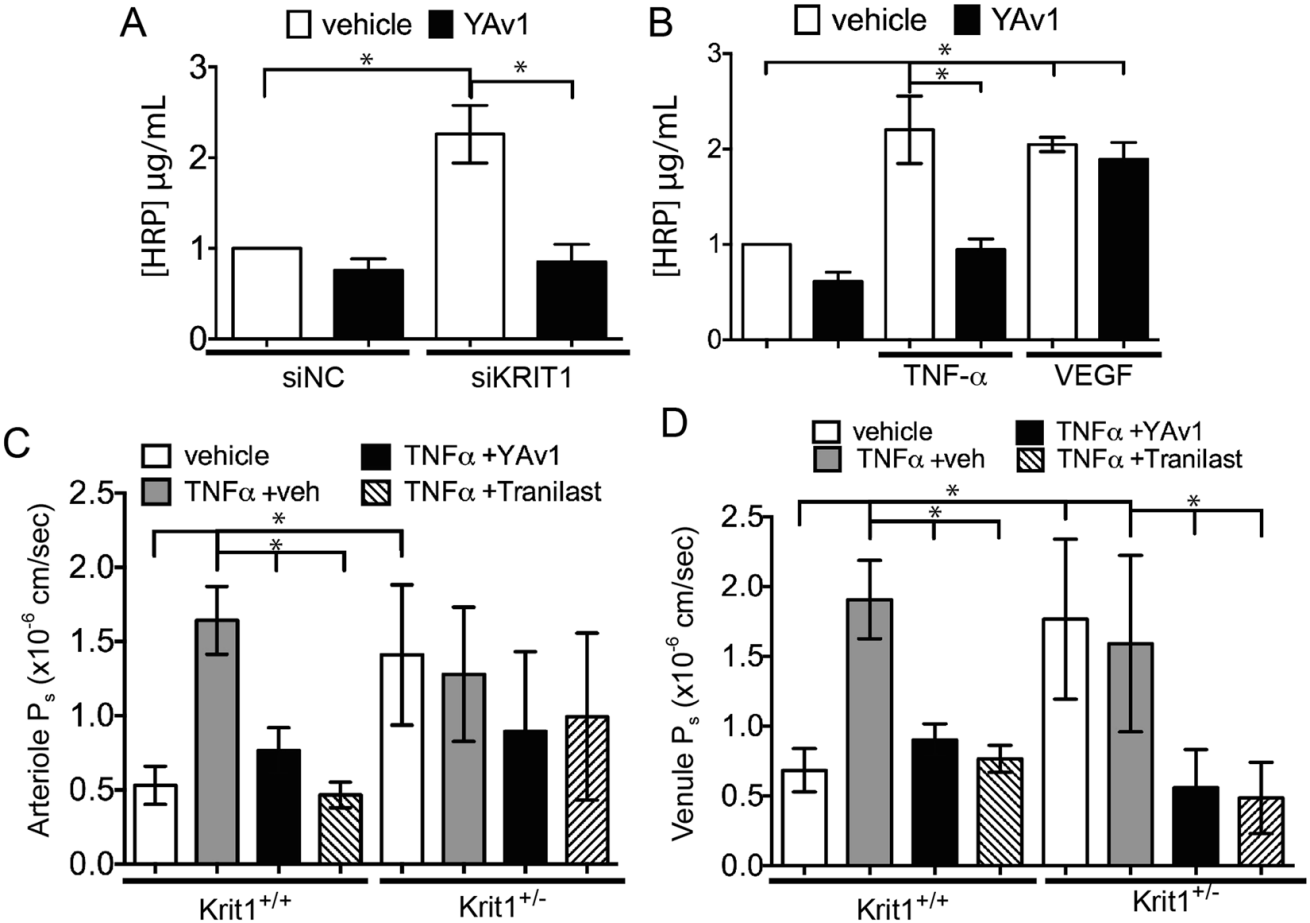
Increased permeability in KRIT1 deficient cells and animals can be reversed by N-(E)-p-coumaroyl-3- hydroxyanthranilic acid. (**A**) HRP leak through HPAEC monolayers transfected with negative control (NC)- or KRIT1-siRNA +/− 100 µM YAv1. Data shown are the mean HRP concentration, +/−SEM; n = 4. p < 0.001 by ANOVA; *p < 0.05 by Tukey’s post-hoc test. (**B**) HRP leak through HPAEC monolayers treated with 25 ng/ml TNF-α or recombinant human VEGF (50 ng/ml) +/− 100 µM YAv1. Data shown are the mean HRP concentration, +/− SEM; n = 4. p < 0.001 by ANOVA; *p < 0.05 by Tukey’s post-hoc test. (**C**) and (**D**) Cremaster arteriole (**C**) and venule (**D**) permeability in wild type (Krit1^+/+^) or heterozygous (Krit1^+/−^) mice treated with TNF-α + vehicle, TNF-α + YAv1, or TNF-α + Tranilast. Data shown are the mean P_s_ +/− SEM. n = 9 vessel sites; p = 0.0002 by non-parametric ANOVA; *p < 0.05 by Dunn’s post-hoc test.

As YAv1 exhibited both antioxidant and anti-NF-κB effects, we wondered whether this compound would affect the KRIT1 depletion-dependent increase in endothelial permeability. Endothelial cell monolayers were transfected with control or anti-KRIT1 siRNA, then treated with either vehicle, YAv1, or YAv1 + TNF-α. As an additional control, we also treated cells with 50 ng/ml VEGF, with and without YAv1. As shown in Fig. 5A, KRIT1 depletion decreased endothelial barrier function (measured by horseradish peroxidase (HRP) leak assay), which was rescued by treatment with YAv1 (siKRIT1, 2.26 +/− 0.32 vs. siKRIT1 + YAv1, 0.85 +/− 0.19). Both TNF-α and VEGF treatment also decreased barrier function in HPAEC cells. However, while YAv1 reversed TNF-α-mediated permeability, it had no effect on VEGF-stimulated permeability (Fig. 5B). To test the effectiveness of YAv1 *in vivo*, wild type (Krit1^+/+^) and Krit1^+/−^ mice were treated with TNF-α, followed by either YAv1, vehicle, or the commercial avenanthramide-mimic Tranilast^29^, prior to measurement of vessel permeability by intravital microscopy. In wild type (Krit1^+/+^) animals, YAv1 and Tranilast blocked TNF-α-induced arteriole and venule permeability (Fig. 5C,D). However, in KRIT1 deficient animals (Krit1^+/−^), YAv1 and Tranilast only partially reduced arteriole permeability (TNF-α + veh 1.28 +/− 0.16, TNF-α + YAv1 0.89 +/− 0.17, TNF-α + Tranilast 0.99 +/− 0.2, Fig. 5C), yet were able to inhibit venular permeability (TNF-α + veh 1.59 +/− 0.22, TNF-α + YAv1 0.56 +/− 0.09, TNF-α + Tranilast 0.49 +/− 0.1, Fig. 5D), similar to the effects of PECAM-conjugated antioxidant enzymes (Fig. 1A,B).

## Discussion

The regulation of vascular permeability is most often considered in the context of increased passage of water and plasma proteins across the vascular wall (edema) and leukocyte extravasation, processes involved in the inflammatory response. Our previous study suggested that the modification of the vascular barrier following decreased KRIT1 expression is independent from inflammatory factors, yet these changes can modify the vascular response to inflammatory mediators. On the other hand, previous findings showed that loss of KRIT1 increases ROS levels in endothelial cells and suggested that KRIT1 expression could modulate the molecular machinery that limit ROS accumulation^12, 18^. As ROS are known to contribute to vascular permeability and are involved in inflammatory signaling, the goal of this study was to investigate this linkage in our model system.

We have now established that ROS signaling is a major contributor to increased endothelial permeability in KRIT1 deficient animals. Perhaps more importantly, we have demonstrated that ROS scavengers targeted specifically to the endothelium can block the increase in vascular permeability in KRIT1 deficient animals. These data agree with previous studies using Tempol and vitamin D3, both pleiotropic intracellular antioxidants, which increased endothelial barrier function in CCM2 deficient cells^30^. Our findings suggest that specifically targeting endothelial cells is as effective as systemic intervention, and that increased superoxide/H_2_O_2_ is responsible for the observed enhanced permeability. Furthermore, we have demonstrated that a newly developed antioxidant and NF-κB inhibitor, yeast avenanthramide I (YAv1), is also capable of limiting vascular permeability in the absence of KRIT1 expression. This molecule shows a strong structural similarity to avenanthramides^19^, a group of anti-inflammatory compounds found in oats, which have been shown to inhibit IL-1β-induced NF-κB activity in endothelial cells^31^ and IL-8-induced NF-κB activation in keratinocytes^32^. Intriguingly, it was previously observed that cell treatment with YAv1 can revert molecular phenotypes caused by KRIT1 loss-of-function, including the down-regulation of FoxO1 and SOD2^19^. Overall, these findings provide important insight into the functional mechanisms behind KRIT depletion-dependent increased permeability and into the role of KRIT1 in maintaining vascular quiescence.

It has long been held that post-capillary venules are the major site for fluid exchange, though more recently it has been accepted that permeability/filtration occurs throughout the microcirculation, including in arterioles. Mouse models suggest CCM lesions arise from capillaries and veins^33^, though this specificity is unverified in human patients. In our study, venule permeability was exquisitely dependent on ROS; indeed, ROS scavenging dampened venular permeability completely in both wild type and KRIT1 deficient animals (Fig. 1), and in the presence and absence of TNF-α. The baseline permeability of wild type arterioles, on the other hand, was unchanged by treatment with PECAM-AOE (Fig. 1C), though the increased baseline permeability in KRIT1 deficient arterioles was reversed (Fig. 1A). Tellingly, TNF-α-induced arteriole permeability was insensitive to ROS scavenging in either genotype, suggesting that permeability regulation downstream of TNF-α occurs through ROS-independent and ROS-dependent pathways (in arterioles and venules, respectively), and highlighting the heterogeneous behavior of endothelial cells from different vascular beds. Thus, while the mechanisms that govern arteriole vs. venule permeability *in vivo* remain ill-defined, our data suggest that venule permeability may be more sensitive to regulation by ROS, which is consistent with several other studies^34, 35^.

We recently discovered that loss of KRIT1 affected the ability of TNF-α to increase permeability, but not to stimulate leukocyte activation^2^. Here, we found that TNF-α could not increase ROS levels in KRIT1 deficient cells (Fig. 2), suggesting that a component of the TNF-α–driven ROS production pathway is affected by the loss of KRIT1, an idea supported by the fact that antioxidant treatment could restore responsiveness to TNF-α in arterioles (Fig. 1A). Subsequently, we found that loss of KRIT1 increased Nox4 expression and up-regulated NF-κB activity (Figs 3 and 4), pathways known to be required for the endothelial response to TNF-α. The connection between TNF-α signaling and ROS is well established, though not well understood. TNF-α receptor activation leads to increased ROS production through activation of Nox2 and Nox4^36^, though the exact mechanism(s) remains unclear. Notably, ROS are also generated downstream of VEGF/VEGFR2 stimulation^37^. We recently reported that KRIT1 deficient cells secrete VEGF and exhibit constitutive activation of VEGFR2^38^. However, in our current study, the antioxidant and NF-κB inhibitor YAv1 was able to block endothelial leak in KRIT1 deficient cells (Fig. 5A), but not in VEGF-treated wild type cells (Fig. 5B). As VEGF increases endothelial ROS, but does not stimulate NF-κB activation, this suggests that the anti-NF-κB function of YAv1 may be more important than its antioxidant activity in the context of permeability regulation. Alternatively, these data could suggest that although the expression of VEGF by KRIT1 deficient cells contributes to the loss of barrier function, it is unlikely that it acts via increased ROS.

Nevertheless, the up-regulation of ROS following TNF-α stimulation is considered a critical component of the endothelial response to TNF-α, and has been suggested to lie upstream of both TNF−α induced increased permeability and NF-κB activation^39, 40^. A substantial portion of ROS production downstream of TNF-α occurs via NADPH oxidase activity, and most studies of ROS generation in response to TNF−α have focused on the activity of Nox2. Nox2, normally expressed at low levels in quiescent endothelial cells, is stimulated by several stimuli, including TNF-α, which increase Nox2 expression or regulate the association of the Nox2 enzyme (gp91phox) with several regulatory subunits (p40, p47 and p67phox, and Rac1)^16^. In contrast, how TNF-α stimulation increases Nox4 activity remains largely unknown. Nox4 does not associate with the regulatory subunits that regulate the other Nox isoforms. Nox4 is thought to be regulated mainly at the transcriptional level, and several mechanisms have been implicated in controlling Nox4 expression, including NF-kB transcriptional activity^16, 41^.

As both KRIT1 depletion and TNF-α increase expression of Nox4, it is possible that they act via the same mechanism, and that this contributes to the lack of permeability response to TNF-α in KRIT1 deficient animals. Herein, we reported an increase in Nox4 protein levels in HPAEC, an increase in Nox4 mRNA expression in Krit1^ECKO^ mouse lung tissue, and an increase in Nox4 staining in the endothelium of CCM-like vascular lesions (Fig. 3). This was accompanied by an increase in NF-κB (p65) expression and reporter activity (Fig. 4). These data suggest that the increase in Nox4 could be mediated transcriptionally by increased NF-κB activation. However, knockdown of KRIT1 in MEFs did not consistently increase Nox4 mRNA (Fig. 3B), while it did stimulate an increase in Nox4 protein in these cells (Fig. 3D,E). Thus, these data do not completely support a transcriptional mechanism for increased Nox4 in KRIT1 depleted cells. Moreover, inhibition of NADPH oxidases by VAS2870 was able to block KRIT1 depletion-dependent NF-κB reporter activity (Fig. 4C), which suggests that conversely, NF-κB may be downstream of Nox4. Both of these signaling relationships have precedence in the literature^42–46^. Therefore, given the lack of clarity regarding Nox4 regulation, and the large number of transcriptional and non-transcriptional signaling pathways active in KRIT1 depleted cells, additional studies to examine how loss of KRIT1 increases Nox4 expression will be required.

These data have clear implications for our understanding of vascular biology and the inflammatory response, yet, as KRIT1 is linked to an inheritable genetic disorder, the implications for the pathogenesis of CCM and for potential therapeutic development should also be considered. CCM lesions may remain clinically silent or unpredictably result in various clinical signs and symptoms at any age^47^. These lesions may be either single or multiple (even hundreds) with variable size (from a few millimeters to a few centimeters). The pleiotropic effects of ROS signaling in the endothelium suggest that oxidative stress could contribute to the development and progression of CCM disease, putatively contributing to several phenotypic characteristics including destabilization of endothelial cell-cell junctions, increased vascular permeability, and altered inflammatory responses^48^. Consistently, recent studies by Choquet *et al*. showed that genetic variants of modifier genes related to oxidative stress and inflammatory responses are associated with phenotypic markers of CCM disease severity^49, 50^, suggesting that inter-individual variability in susceptibility to either oxidative stress or inflammation may contribute to CCM disease pathogenesis.

As we noted in our introduction, recent discoveries based on cell and animal models of KRIT1, CCM2, and CCM3 protein deficiency have highlighted a wide, interconnected network of signaling pathways that are regulated by these proteins. Interestingly, several of these mechanisms are regulated by- or regulate- cellular redox signaling^48^. Increased ROS production downstream of loss of KRIT1 causes the up-regulation of c-Jun, a basic component of the redox-sensitive transcription factor AP-1, in both cells and human CCM tissue samples^18^. ROS can also stimulate the activation of the TGF-β pathway, which has been observed in KRIT1 knockout mice^51^, and may be related to the up-regulation of c-Jun transcriptional activity^52^. Mitogen-activated protein kinase kinase kinase 4 (MEKK4) and Kruppel-like transcription factors (KLF) are both known targets and regulators of redox signaling^53–55^, as is Toll-like receptor 4 (TLR4)^56^, suggesting that ROS signaling may also be involved in the MEKK4/KLF4/TLR4 signaling axis recently identified as important in CCM pathogenesis^57, 58^. Recent findings have also shown that KRIT1 deficient cells are defective in autophagy^59^, which is up-regulated in response to oxidative stress^60, 61^. Thus, KRIT1 deficiency may lead to alterations in both ROS generation, as we show in this study, and in the adaptive response to increased oxidative stress.

It follows that targeting ROS/redox signaling may be a viable treatment strategy for CCM. Given that antioxidant treatment was particularly effective in venules, where CCM lesions develop^33^, our data supports the idea that controlling the redox state at the blood brain barrier could be a promising therapy for CCM. However, as studies in other redox-sensitive conditions have shown, reducing ROS generation below a certain threshold can be deleterious. In addition, though we have shown that antioxidant treatment is effective at reducing the increase in endothelial permeability, this may not be sufficient to prevent lesion formation. However, Gibson *et al*. reported that treatment with the antioxidants Tempol and vitamin D3 were able to reduce lesion burden in CCM2 deficient mice^30^. As a result, additional studies to examine the effectiveness of antioxidant treatment on reducing lesion formation in KRIT1 null animals, including the targeting of antioxidant treatment to the endothelium, are warranted.

In sum, our findings strongly support a role for increased ROS signaling in promoting increased baseline permeability in the absence of KRIT1. The increase in ROS likely prevents TNF-α from further increasing permeability in KRIT1 deficient cells and animals. This implies that the mechanism by which KRIT1 increases ROS is likely also utilized by TNF-α, which will help guide our future studies. As KRIT1 increased Nox4 expression and NF-κB activity, the nexus of Nox4, NF-κB and TNF-α signaling is a good starting point. Finally, we demonstrated that limiting ROS levels, either by introducing ROS scavenging enzymes into the blood, or by treating with recombinant yeast avenanthramide I, was able to block KRIT1 depletion-dependent increased permeability *in vitro* and *in vivo*, thus supporting the idea that antioxidant therapy could be a potential treatment for CCM.

## Methods

### Reagents

Conjugation via amino chemistry was used to prepare anti-PECAM/enzyme conjugates as described previously^62^. A rat monoclonal antibody against murine PECAM (clone MEC- 13.3; BD Biosciences, San Jose, CA) was conjugated with catalase or SOD at 1:2 IgG to enzyme molar ratio, respectively. The effective diameter of the obtained conjugates was measured by a dynamic light-scattering apparatus (90Plus Particle Sizer). Conjugates in 7% sucrose were frozen by liquid nitrogen and stored at −80 °C until use. Recombinant mouse TNF-α, VAS2870, and Tranilast (2-([(2*E*)-3-(3,4-dimethoxyphenyl)prop-2- enoyl]amino)benzoic acid, Rizaben ®) were obtained from Sigma-Aldrich (St. Louis, MO). N-(E)-p-coumaroyl-3- hydroxyanthranilic acid (Yeast avenanthramide I, YAv1) was prepared and validated as described in ref. 19.

### Mice

KRIT1 heterozygous (*Krit1* ^+/−^) mice were obtained from Dr. Doug Marchuk (Duke University). These mice are on a clean C57BL/6 background after having been backcrossed 10 generations to C57BL/6NCrl. A conditional endothelial KRIT1 knockout mouse (PDGFBiCreER^T2^Krit1^flox/flox^) was obtained from Dr. Dean Li (University of Utah). In these mice, tamoxifen injection stimulates PDGFB promoter-driven Cre-ER activity, causing the deletion of exons 4–8 of KRIT1. In this study, PDGFBiCreER^T2^Krit1^flox/flox^, and PDGFBiCreER^T2^Krit1^flox/+^ animals were treated with tamoxifen at P0 as in ref. 6 to yield endothelial KRIT1 null (*Krit1* ^*ECKO*^) and heterozygous (*Krit1* ^+/−^) control mice. Mouse lung tissue was harvested from both male and female mice at 4 months. Lung tissue was used instead of cremaster muscle as this tissue is enriched in endothelial cells, thus eliminating the overwhelming contribution of mRNA from skeletal muscle cells present in the cremaster. All mice were bred and maintained under standard conditions in the University of Rochester animal facility, which is accredited by the American Association for Accreditation of Laboratory Animal Care. All protocols were approved by the University of Rochester Committee on Animal Resources and all experiments were performed in accordance with relevant guidelines and regulations.

### Intravital Microscopy

Mice were used between 8 and 12 weeks of age; littermate controls were used in all experiments. Male wild type (*Krit1* ^+*/*+^) or KRIT1 heterozygous (*Krit1* ^+/−^) mice were anesthetized with sodium pentobarbital (65 mg/kg i.p.) and maintained on supplemental anesthetic via a jugular catheter. Preparation and visualization of the cremaster muscle, as well as measurement of permeability to Alexa-488 BSA was performed as previously described^2^. The vascular wall P_s_ (cm/sec) was calculated from the measured flux per unit microvessel surface area at a known BSA concentration gradient^63^, with corrections for the source volume and surface area due to the confocal slice^64^. As ROS regulate arteriole tone, which has profound effects on permeability, we excluded from our analysis vessels that had a significant change in diameter during the measurement, which comprised <5% of all imaged vessels. A 1:1 mixture of anti-PECAM conjugated SOD and anti-PECAM conjugated catalase (100 µg each in 100 µl) was injected via tail vein 1hr before surgery. Yeast-avenanthramide I (45 mg/kg) and Tranilast (40 mg/kg) were administered by oral gavage in 15% ethanol 1hr prior to imaging. Images were acquired from at least 10 vessel sites, which are the appropriate determinant for n in these experiments. Data were given as arteriole and venule means +/− SEM.

### Cell Culture and Transfection

Human pulmonary artery endothelial cells (HPAEC, Invitrogen/ThermoFisher, Waltham MA) were cultured in 1:1 DMEM:F/12, supplemented with 5% fetal bovine serum (FBS), 1% endothelial cell growth supplement (ECGS, ScienCell, Carlsbad, CA), 1% antimyotic/antibiotic solution, and 50 µM heparin, at 37 °C with 5% CO_2_. HPAEC were grown on 2 µg/cm^2^ gelatin coated tissue culture plates and only passage 3 to passage 6 were used in experiments. KRIT1 knockout (KO) and reconstituted (9/6) MEF were cultured in DMEM high glucose with 10% FBS, 1% penicillin/streptomycin/L-glutamine, and 1% non-essential amino acids, at 37 °C with 5% CO_2_. HPAEC were transfected with 30 ng siRNA using the HiPerfect transfection reagent (Qiagen, Valencia, CA) as reported previously (14). Alternatively, HPAEC were transfected with siRNA using siPort Amine (Ambion/Invitrogen), according to manufacturer’s instructions.

Non-targeting negative control siRNA #1 and anti-KRIT1 siRNA (AM16708, Ambion/Invitrogen) were used as reported previously^1^. Knockdown efficiency was measured in each experiment by qPCR detection of KRIT1, as indicated in the appropriate figures. Transfection efficiencies ranged from 80–95% based on transfection of fluorescently labeled siRNAs (data not shown). Activity of this anti-KRIT1 siRNA against human protein has been reported previously^1^. Co-transfection of siRNA and cDNA was performed using Amaxa nucleofection, as previously described^4^.

### RNA isolation and semi-quantitative RT-PCR

RNA was isolated using an RNeasy Purification Kit (Qiagen) or Trizol extraction according to the manufacturer’s instructions. Complementary DNA was obtained with Transcriptor reverse transcriptase (Roche, Indianapolis, IN) using oligo dT primers. Amplifications were run in a 7000 real-time PCR system (Applied Biosystems/ThermoFisher) using mouse primer sets (Supplemental Information Table 1, and ref. 4) or Taqman gene expression assays (*Krit1*, Hs00184988; *Gapdh*, Hs99999905; *Nox2* (*CYBB*), Hs00166163; *Nox4*, Hs01379108; ThermoFisher). Each value was calculated using the comparative Ct method^65^ and normalized to *Gapdh* internal control. All samples were run in at least triplicate.

### Immunoprecipitation and Western Blotting

Lysates were prepared as reported previously^4^. Nox4 was immunoprecipitated using 2 µg monoclonal anti-Nox4 antibody (Millipore/Sigma). Lysates were probed with polyclonal rabbit anti-Nox4 (Novus Biologicals, Littleton CO) at a dilution of 1:1000. Control lysates were immunoprecipitated with mouse non-immune IgG (Santa Cruz, Dallas, TX). Goat anti-rabbit Dylight-680 and goat-anti rabbit 800 (ThermoFisher) were used as secondary antibodies and the membranes imaged using an Odyssey Infrared Imaging System. NF-κB p65 was detected using a rabbit polyclonal anti-p65 (C-20) antibody from Santa Cruz. Tubulin was detected as a loading control (monoclonal anti-α-tubulin, Sigma). Goat anti-rabbit and goat anti-mouse HRP were used as a secondary antibodies and the blot was developed using Luminata™ Forte Western HRP substrate reagent, then exposed to film.

### Immunohistochemistry

Immunohistochemical staining of Nox4 was performed on cross-sections of paraffin-embedded mouse brain tissue. Antigen retrieval was performed by incubation in citrate buffer at 100 °C for 10 min. Slides were treated with 3% H_2_O_2_ for 5 min to remove endogenous peroxidases, then blocked for 1hr in a solution of 2% normal goat serum, 1% BSA and 0.01% Triton X-100. Rabbit Nox4 polyclonal antibody was added at a 1:100 dilution in blocking buffer and slides were incubated 4hr at room temperature in a humidified chamber. Control serial sections were incubated with an equivalent concentration of normal rabbit IgG. Donkey anti-rabbit IgG-HRP (1:250) was used as secondary antibody. Staining was developed using the DAB + liquid stain reagent (diaminobenzidine, Dako, Denmark); slides were counterstained for 30 sec. with Mayer’s hematoxylin.

### Endothelial Monolayer Leak Assay

HPAECs were plated onto 3μm pore polyester transwell filters coated with 10 µg/ml human plasma fibronectin (gift from Dr. Denise Hocking, University of Rochester). Cells were transfected with siRNA 24 hours after plating and grown for 72 hours at 37 °C to full confluence. Cells were then incubated in DMEM with 0.5% FBS for 2 hours, then treated with or without YAv1 (100 µM) or TNF-α (25 ng/ml) for 24hrs, or with VEGF (50 ng/ml) for 40 minutes. Horseradish peroxidase (HRP, 1.5 µg/ml, Sigma) was added to upper wells, and the plates were then incubated for an additional 2 hours at 37 °C. The HRP content of the lower chamber medium was then measured using a 3,3′, 5,5′-tetramethylbenzidine colorimetric assay as described in ref. 38.

### NFκB reporter

Reporter assays were performed using the Dual-Glo luciferase assay system (Promega, San Luis Obispo, CA) according to the manufacturer’s instructions. Firefly luciferase activity produced by the NFκB-dependent firefly luciferase reporter plasmid pNF-κB -Luc (Stratagene, La Jolla, CA) was measured using a Synergy H4 hybrid plate reader. All transfections included the constitutive Renilla luciferase plasmid pRL-TK, which was used to normalize for transfection efficiency. As appropriate, cells were treated with 25 ng/mL TNF-α for 4 hours prior to luciferase detection.

### DHE/CellROX staining

Qualitative measurement of intracellular ROS levels was performed using a well-established method based on the cell-permeable ROS-sensitive fluorogenic probe dihydroethidium, which has a sensitivity for superoxide anions. Briefly, cells grown to confluence in complete medium were washed twice with PBS, incubated with DHE at a final concentration of 5 mM in PBS at 37 °C for 20 min, treated +/− 25 ng TNF-α for 30 min, then fixed and analyzed by fluorescence microscopy on an Olympus IX70 fluorescence microscope equipped with a Hamamatsu CCD camera and MetaMorph software for hardware control and image acquisition. Quantitative analyses were performed using the CellROX®Green Reagent (ThermoFisher) following the manufacturer’s recommended protocol.

### Statistics

Statistical analysis, including ANOVA (with appropriate post-hoc test for multiple comparisons), t-test, and Shapiro-Wilk test for normality was performed using PRISM software (version 4.0, GraphPad Software Inc., La Jolla, CA). *In vivo* permeability measurements exhibit a non-Gaussian distribution, therefore these data were analyzed by ANOVA using the Kruskal-Wallis correction and Dunn’s post-hoc test. All other comparisons were analyzed by ANOVA and Tukey’s post-hoc test, or by two-tailed t-test as indicated in the figure legend. Significance for all analyses was set at α = 0.05.

### Data Availability

The datasets generated during and/or analyzed during the current study are available from the corresponding author on reasonable request.

## Electronic supplementary material

Supplemental Information
