# Supplementary material for: Up-regulation of NADPH oxidase-mediated redox signaling contributes to the loss of barrier function in KRIT1 deficient endothelium
Source: Sci Rep. 2017 Aug 15;7:8296. doi: 10.1038/s41598-017-08373-4 (PMC5558000; doi:10.1038/s41598-017-08373-4)
Supplement: Supplementary file 1 — Supplemental Information [file 41598_2017_8373_MOESM1_ESM.pdf]

Up-regulation of NADPH oxidase-mediated redox signaling contributes to the loss of barrier function in KRIT1 deficient endothelium

Luca Goitre[1], Peter V. DiStefano[2], Andrea Moglia[3], Nicholas Nobiletti[2], Eva Baldini[2,4], Lorenza Trabalzini[4], Julie Keubel[2], Eliana Trapani[1], Vladimir V. Shuvaev[5], Vladimir R. Muzykantov[5], Ingrid H. Sarelius[2], Saverio Francesco Retta[1], Angela J. Glading[2]

Supplemental Information

Gene	Forward primer	Reverse Primer	Species
SOD2	*gta gta agc gtg ctc cca cac*	*gac cca ttg caa gga acaa*	mouse
Nox2 (Cybb)	*gag gtt ggt tcg ctt ttg gc*	*cag gag cag agg tca gtg tc*	mouse
Nox4	*ggg tgt gca gag aca tcc aa*	*gac gcc caa tga aaa gtc tcg*	mouse

Table S1. Primer sets for RT-PCR analysis.
